# Linkage Specific Fucosylation of Alpha-1-Antitrypsin in Liver Cirrhosis and Cancer Patients: Implications for a Biomarker of Hepatocellular Carcinoma

**DOI:** 10.1371/journal.pone.0012419

**Published:** 2010-08-25

**Authors:** Mary Ann Comunale, Lucy Rodemich-Betesh, Julie Hafner, Mengjun Wang, Pamela Norton, Adrian M. Di Bisceglie, Timothy Block, Anand Mehta

**Affiliations:** 1 Department of Microbiology and Immunology, Drexel Institute for Biotechnology and Virus Research, Drexel University College of Medicine, Doylestown, Pennsylvania, United States of America; 2 Division of Gastroenterology and Hepatology, Saint Louis VA Medical Center, Saint Louis University School of Medicine, St Louis, Missouri, United States of America; Yonsei University, Republic of Korea

## Abstract

**Background:**

We previously reported increased levels of protein-linked fucosylation with the development of liver cancer and identified many of the proteins containing the altered glycan structures. One such protein is alpha-1-antitrypsin (A1AT). To advance these studies, we performed N-linked glycan analysis on the five major isoforms of A1AT and completed a comprehensive study of the glycosylation of A1AT found in healthy controls, patients with hepatitis C- (HCV) induced liver cirrhosis, and in patients infected with HCV with a diagnosis of hepatocellular carcinoma (HCC).

**Methodology/Principal Findings:**

Patients with liver cirrhosis and liver cancer had increased levels of triantennary glycan-containing outer arm (α-1,3) fucosylation. Increases in core (α-1,6) fucosylation were observed only on A1AT from patients with cancer. We performed a lectin fluorophore-linked immunosorbent assay using *Aleuria Aurantia* lectin (AAL), specific for core and outer arm fucosylation in over 400 patients with liver disease. AAL-reactive A1AT was able to detect HCC with a sensitivity of 70% and a specificity of 86%, which was greater than that observed with the current marker of HCC, alpha-fetoprotein. Glycosylation analysis of the false positives was performed; results indicated that these patients had increases in outer arm fucosylation but not in core fucosylation, suggesting that core fucosylation is cancer specific.

**Conclusions/Significance:**

This report details the stepwise change in the glycosylation of A1AT with the progression from liver cirrhosis to cancer and identifies core fucosylation on A1AT as an HCC specific modification.

## Introduction

Infection with hepatitis B virus (HBV) or hepatitis C virus (HCV) is the major etiology of hepatocellular cancer (HCC) [1]–[4]. Both HBV and HCV cause acute and chronic liver infections, and most chronically infected individuals remain asymptomatic for many years [5]. About 10% to 40% of all chronic HBV carriers eventually develop liver cancer, and it is estimated that over one million people worldwide die because of HBV- and HCV-associated liver cancer [2], [6], [7]. Indeed, HBV and HCV infections are associated with over 80% of all cases of HCC worldwide and can be as high as 96% in regions where HBV is endemic [3].

The progression of liver disease to liver cancer is primarily monitored by serum levels of the oncofetal glycoprotein, alpha-fetoprotein (AFP), or the core fucosylated glycoform of AFP, AFP-L3. AFP can, however, be produced in many circumstances, including in relation to other liver diseases [8]–[10] and is not present in all those with HCC [11]. Therefore the use of AFP as a primary screen for HCC has been questioned [12], and more sensitive serum biomarkers for HCC are needed.

The glycosylation of proteins is cell specific. The N-linked glycosylation of a protein reflects modifications that occurred in the cell from which it came [13]. The glycosylation of the same protein secreted from diseased tissue, malignant cells or normal cells may, and often do, differ [14]. We, and others, have observed changes in N-linked glycosylation with the development of cirrhosis and HCC [15]–[19]. Specifically, the amount of core fucosylated N-linked glycan derived from total protein preparations isolated from the serum of individuals chronically infected with HCV and from those with a diagnosis of HCC was consistently greater than that in healthy patients or in those with HCV and “inactive” disease [19].

Using fucose-specific lectins to identify the proteins that become fucosylated in patients with liver disease, we identified more than 100 glycoproteins from patients with HCC and/or cirrhosis that contained increased fucosylation [19]. One of these proteins was alpha-1-antitrypsin (A1AT). We analyzed the N-linked glycosylation of the five major isoforms of A1AT and discovered, in addition to increased levels of core fucosylation, significant increases in outer arm fucosylation with the development of liver cancer. Using a lectin-based assay, we measured this change in over 400 patients with liver disease and found AAL reactive A1AT could detect HCC with a sensitivity of 70% and a specificity of 86% using a cut-off of 5 relative units. Glycan analysis of the false positives identified outer-arm fucosylation as being the cause of false positivity. In contrast, increases in core fucosylation were found only in patients with cancer. The reasons for this change and the clinical usefulness of this change are discussed.

## Materials and Methods

### Ethics Statement

Both the Drexel University College of Medicine and the Saint Louis University Institutional Review Boards approved the study protocol, which was consistent with the standards established by the Helsinki Declaration of 1975. Written informed consent was obtained from each participant.

### Patients

Serum samples were obtained from the Saint Louis University School of Medicine (Saint Louis, MO). Demographic and clinical information along with a blood sample was collected from each participant in a serum separator tube. The sample was spun within 2 hours, and the serum was stored at –80°C until testing. Patients were enrolled in the Saint Louis University Liver Cancer Clinic and HCC were diagnosed using the same criteria established for the HALT-C trial [20]. Participants had HCC identified by biopsy, by a new hepatic defect showing vascular enhancement on one imaging modality (ultrasound [US], magnetic resonance imaging [MRI], or computed tomography [CT]) with AFP levels >1000 ng/ml, or by presumed HCC. Participants were presumed to have HCC if they had a discrete hepatic defect on US with AFP levels <1000 ng/ml and either two other scans (MRI, CT, angiography) indicating malignancy with at least one of the following characteristics: hypervascularity, arterial to portal vein shunts, portal vein thrombosis near the defect, tumor in the portal vein, or one other scan (MRI or CT) showing features characteristic of HCC and either an increase in size over time after initial discovery (at least doubling if less than 1 cm) or an increase in the level of AFP to >200 ng/ml. Tumor staging was determined using the United Network of Organ Sharing-modified (UNOS) TNM staging system for HCC. For the cirrhosis group, patients with hepatitis C and biopsy-proven cirrhosis were enrolled. All cirrhotic controls were screened for HCC using US, CT, or MRI prior to enrollment. Patients who were HBsAg+ (with or without HBV DNA were classified into the HBV group. Similarly, patients who were HCV RNA positive, with no evidence of cirrhosis, were classified into the HCV group. Patients with other non viral liver disease but without cirrhosis were classified into the other liver disease group (OLD). Control patients were healthy patients recruited by the study to act as controls.

### Two-Dimensional (2-D) Gel Electrophoresis

A total of 7 µl of serum was diluted in 330 µl of solubilization buffer containing 7 M urea, 2 M thiourea, 4% CHAPS, 65 mM DTT, 5 mM tributylphosphine, and a 0.4% mixture of carrier ampholytes (Servalyt pH 2–4, pH 3–10, pH 9–1, 1∶2∶1). Samples were vortexed periodically for 1 h and applied to an 18-cm pH 3-7 NL immobilized pH gradient (IPG) strip (Amersham, Piscataway, NJ, USA). Gel rehydration was carried out for 14 h at 50 V and focused using the IPGPhor (Amersham) isoelectric focusing apparatus. After focusing, gel strips were first reduced then alkylated in 6M urea, 2% SDS, 30% glycerol, 50 mM Tris, pH 6.8, and either 30 mM DTT or 75 mM iodoacetamide for 10 min each. The second dimension was resolved with an 8% to 18% acrylamide–0.8% PDA gradient gel on a Protean II xi Cell (Biorad Laboratories Headquarters, Hercules, CA, USA) with the running conditions set to 20 mA/gel for 20 min and 40 mA/gel for 4 h. Gels were fixed and stained with colloidal Coomassie. Gels were imaged using the Odyssey Infrared Imaging System (Li-Cor Biosystems, Lincoln, Nebraska) and analyzed using NonLinear Dynamics Progenesis Workstation gel imaging software (NonLinear Durham, NC, USA).

### Matrix-Assisted Laser Desorption/Ionization-Time of Flight (MALDI-TOF) Mass Spectrometry

Protein spots were excised from colloidal Coomassie blue stained gels, destained, and digested with trypsin. Recovered peptides were concentrated and desalted using ZipTip C18 (Millipore, Bedford, MA, USA) according to the manufacturer's directions and prepared for MALDI-TOF mass spectrometry by mixing 0.5 mL of peptide mixture with 0.5 mL of 10 mg/mL α-cyano-4-hydroxycinnamic acid and 1% formic acid in 50% acetonitrile and allowing the droplet to dry on the MALDI plate. Peptide mass maps were obtained using a Voyager-DE Pro Mass Spectrometer (Applied Biosystems, Life Biotechnologies, Carlsbad, CA) operated in positive ion reflectron mode. Proteins were identified from the peptide mass maps using the MASCOT online database (www.matrixscience.com) to search the nonredundant protein database.

### Glycan Analysis

The 2DE gel spots were excised and destained using 40% methanol 7% acetic acid. The gel plugs were dehydrated with acetonitrile, the acetonitrile was removed and 25mM DTT was allowed to absorb into the plug. The plug was heated to 100°C for 5 min, allowed to cool, and alkylated in the dark for 30 min with 75mM iodoacetamide. The gel plugs were washed using two repeats of acetonitrile dehydration and 20 mM ammonium bicarbonate rehydration. After a final dehydration, the gel plugs were dried in a speed vac. Peptide∶N-glycosidase F (PNGase F) was diluted with 20 mM ammonium bicarbonate pH 7 and allowed to adsorb into the gel plug. The gel plug was then covered with the same solution and allowed to incubate overnight at 37°C. The glycans were eluted from the gel plug by sonication in Milli-Q water three times; the elutant was pooled, dried down, and labeled with a 2AB dye (Ludger, Oxford, UK) according to the manufacturer's instructions. The glycans were then cleaned up using paper chromatography and filtered using a 0.22-µm syringe filter. Fluorescently labeled glycans were subsequently analyzed using the Waters Alliance high-performance liquid chromatography system with a normal phase column (TSK amide 80 columns) complemented with a Waters fluorescence detector and quantified using the Millennium Chromatography Manager (Waters Corporation, Milford, MA). The mobile phase consisted of solvent A (50 mM ammonium formate, pH 4.4) and solvent B (acetonitrile). The gradient used was as follows: linear gradient from 20% to 58% solvent A at 0.4 mL/minute for 152 min followed by a linear gradient from 58% to 100% solvent A for the next 3 min. The flow rate was increased to 1.0 mL/minute; the column was washed in 100% solvent A for 5 min. Following the wash step, the column was equilibrated in 20% solvent A for 22 min in preparation for the next sample. Glycan structures were identified by calculating the glucose uptake value and exoglycosidase digestion, as described previously [21].

### Lectin-Fluorophore-linked Immunosorbent Assay (FLISA)

The capture antibody (mouse antihuman A1AT, AbD Serotec, Raleigh NC, USA), was incubated with 10 mM sodium periodate for 1 h at 4°C. This treatment ensures the lectin is unable to react with the glycosylation of the antibody and does not affect antibody substrate binding. An equal volume of ethylene glycol was added, and the oxidized antibody was brought to a concentration of 10 µg/mL with sodium carbonate buffer, pH 9.5. Antibody (5 µg/well) was added to the plate and, following incubation, was washed with 0.1% Tween 20mM phosphate buffered saline pH 7.4. The plate was blocked overnight with 3% bovine serum albumen/phosphate buffered saline. For analysis, 5 µl of serum was diluted in 95 µl blocking reagent in Heterophilic Blocking Tubes^tm^ (Scantibodies Laboratory, Inc. Santee, CA, USA) and was incubated at room temperature for 1 h. Subsequently, samples were added to the plates for 2 h and washed 5 times in lectin incubation buffer (10 mM Tris pH 8.0, 0.15M NaCl, 0.1% Tween 20). Fucosylated A1AT was detected with a biotin conjugated *Aleuria aurantia* lectin (AAL) (Vector Laboratories, Burlingame, CA). Bound lectin was detected using IRDye 800 conjugated streptavidin; the signal intensity was measured using the Odyssey infrared imaging system (LI-COR Biotechnology, Lincoln, Nebraska). In all cases, signal intensities were compared to those of signals detected with commercially purchased human serum (Sigma Chemicals). The lectin-FLISA detects the amount of fucosylation present on an equal quantity of molecules captured from each patient sample and is performed in a manner independent of the total amount of protein in any given patient.

### Statistical Analysis

Descriptive statistics for staged patients were compared by scatter plots that included the outliers. All values were reported as mean values plus or minus the standard error unless otherwise stated. Because the data did not follow typical Gaussian distribution, a nonparametrical test (two-tailed, 95% confidence, Mann-Whitney test) was used to determine statistical difference between the groups. To determine the optimal cutoff value for each marker, the receiver operating characteristic curves were constructed using all possible cutoffs for each assay. The area under the receiver operating characteristic curve was constructed and compared as described previously. A two-tailed P value of 0.05 was used to determine statistical significance. All analyses were performed using GraphPad Prism (San Diego, CA, USA).

## Results

### Levels of A1AT and Degree of Sialyation in Patients with Liver Cirrhosis and HCC

**Figure 1:**
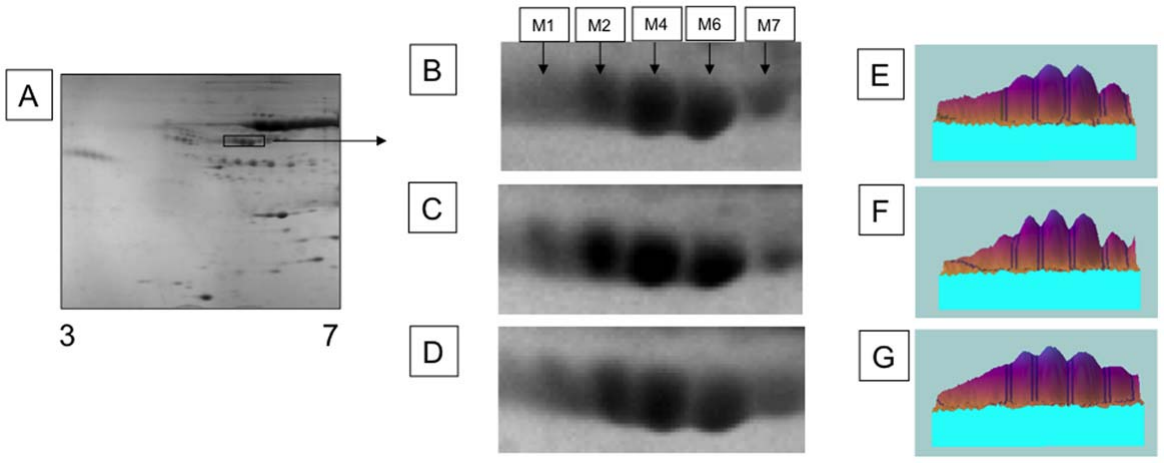
Two-dimensional gel purification of alpha-1-antitrypsin isoforms. Sera from pools of healthy controls (**A**, **B**, **E**) and cirrhosis (**C**, **F**) and cancer (**D**, **G**) patients were focused using IPGPhor 3–7 NL first dimension strips followed by SDS-PAGE separation on 8% to 18% acrylamide gels. The pI of selected gel spots are M1 = 4.91, M2 = 4.95, M4 = 5.00, M6 = 5.05, and M7 = 5.10. Panels E, F, and G show the relative abundance of each gel spot.

Pooled sera from healthy patients (n = 20), patients with liver cirrhosis (n = 20) and patients with HCC with a background of cirrhosis (n = 20) were resolved via 2-D gel electrophoresis (2DE) (Table 1). Figure 1A shows a representative 2-DE of normal human serum and Figures 1B, 1C and 1D show a focus on the 5 isoforms (M1, M2, M4, M6, and M7) of A1AT from the three patient groups. Three major and two minor A1AT isotypes are commonly seen when human serum is isoelectrically focused and run on a 2-D gel [22], [23] and these are not altered in the three patient groups. The A1AT concentrations in each patient group were comparable (Figures 1E–1G) and fell within normal serum concentrations. This result was confirmed by analyzing A1AT an Enzyme-linked immunosorbent assay (ELISA), whereby the A1AT levels were 3.2 mg/mL in the composite from the healthy patients, 3.3 mg/mL in the composite from the cirrhotic patients, and 3.3 mg/mL in the composite from the HCC patients.

**Table 1:** Patients Utilized in Study.

Disease Diagnosis^1^	HCC^2^	Cirrhosis^3^	HBV^4^	HCV^5^	OLD^6^	Controls^7^
Number	63	65	33	215	62	20
Etiology% (HBV/HCV/crypto/alcohol/other)^8^	14/52/6/20/8	N/A	N/A		0/0/15/16/69	N/A
Age	58.04±11	50±8	58.6±12	58±3	51±3	55±8
Gender M∶F%	71∶29	84∶16	75/25	60/40	56∶44	50∶50
MELD Score^9^	11.8±5	N/A	N/A	9±2	N/A	N/A
Child Class (A/B/C/) or NA%^10^	52∶29∶9∶10	88∶8∶4	N/A	N/A	N/A	N/A
Tumor Stage (1/2/3/4) %^11^	26∶48∶12∶14	NA	N/A	NA	N/A	N/A

1 Samples were provided coded from St. Louis University Medical School.

2,3 HCC or cirrhosis was determined by MRI or by liver biopsy.

4 Patients classified as HBV only were defined as those with HBsAg positivity but no evidence of liver cirrhosis.

5 Patients classified as HCV only were defined as those with HCV RNA positivity but no evidence of liver cirrhosis.

6 OLD, including cryptogenic liver disease, alcohol induced liver disease, nonalcoholic steatohepatitis, and autoimmune hepatitis.

7 Patients without any evidence of liver disease were used as controls.

8 Etiology: HBV, hepatitis B virus; HCV, hepatitis C virus; crypto, cryptogenic liver disease; alcohol, alcohol induced liver disease; other, liver disease of unknown origin.

9 MELD: Model for end stage liver disease.

10 The percent of patients with each Child-Pugh score is given as a percentage in each group.

11 Tumor staging was determined using the United Network of Organ Sharing-modified TNM staging system for HCC. The percent of patients within each stage is given. NA, not available.

HBV, hepatitis B virus; HCC, hepatocellular carcinoma; HCV, hepatitis C virus; MELD, model for end stage liver disease; OLD, other liver disease.

We performed glycan analysis on the M1, M2, M4, M6, and M7 isotypes from the pools of sera from normal participants and cirrhotic and HCC patients (Figures 1B–1D) and examined the sialylation patterns of each isotype. Figure S1 shows a representative glycan profile for the M4 isotype from the controls. Consistent with results of previous studies [24], the biantennary glycan is the most abundant species. Figure S1-B shows the level of the sialyated biantennary and triantennary glycan associated with each isoforms from each patient group. As S1-B shows, the level of sialyation on the bi-anntennery glycan (A2G2S1 or A2G2S2) was similar in the different patient groups. Similarly, the level of sialyation was not altered on the triantennary glycan. However, there was an increase in the level of the tri-sialyated α-1,3 linked fucosylated outer arm fucosylated glycan (A3F(3)1G3S3) on A1AT from the cancer patients, as compared to the healthy and cirrhotic patients. These peak are indicated in figure S1A & B with an asterisk.

### Increased Levels of Core and Outer Arm Fucosylation Are Observed on A1AT from Patients with HCC

**Figure 2:**
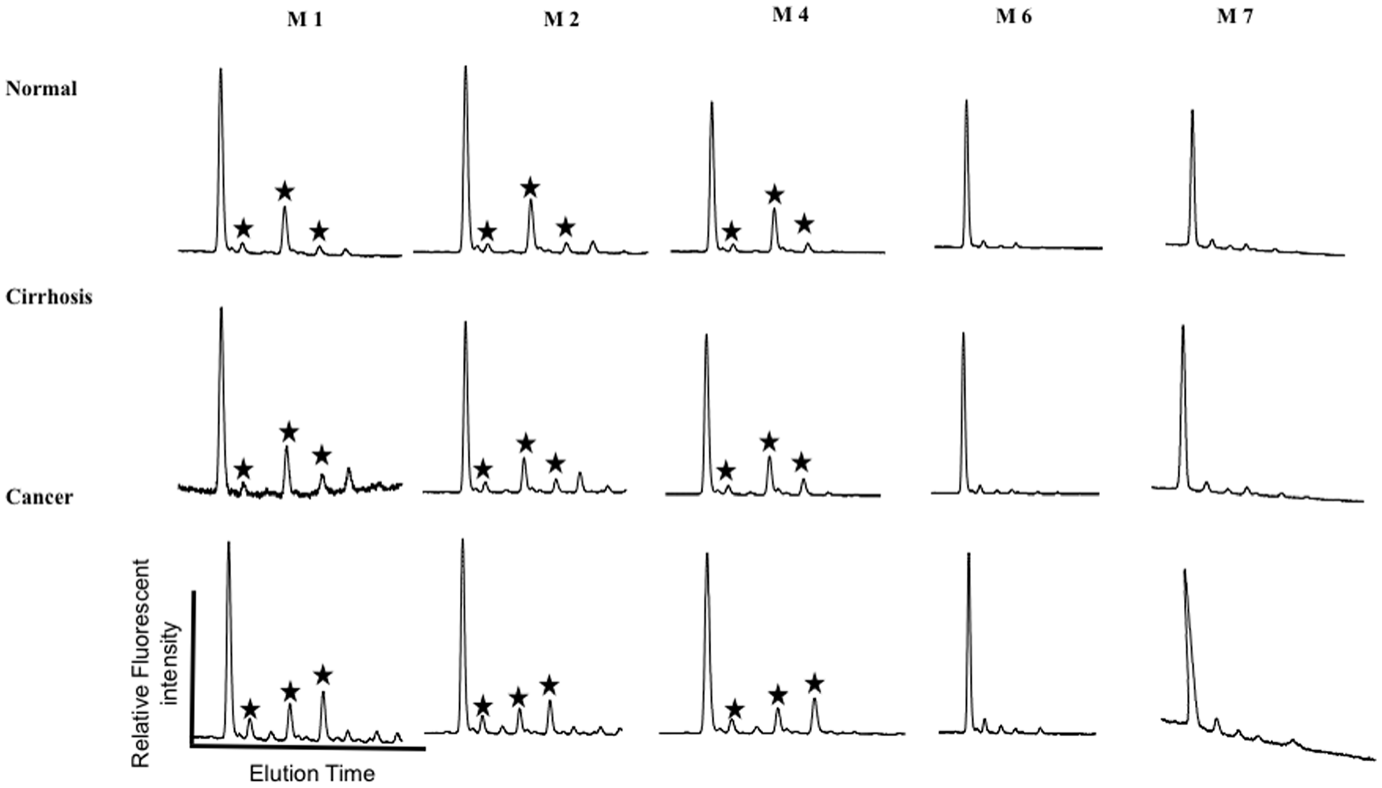
The desialylated N-linked glycan profile for each of the five A1AT isoforms from normal controls (top) and cirrhotic (middle) or HCC patients (bottom). The major peaks that are altered are indicated with an asterisk and are (from left to right) a core fucosylated bianntennary glycan (F96)A2G2), a trianntennary N-linked glycan, and a trianntennary N-linked glycan with a single outer arm fucose residue (A3F[3]3). The percent of each of these peaks in the different isoforms and in the different patient groups is shown in Table 2.

**Figure 3:**
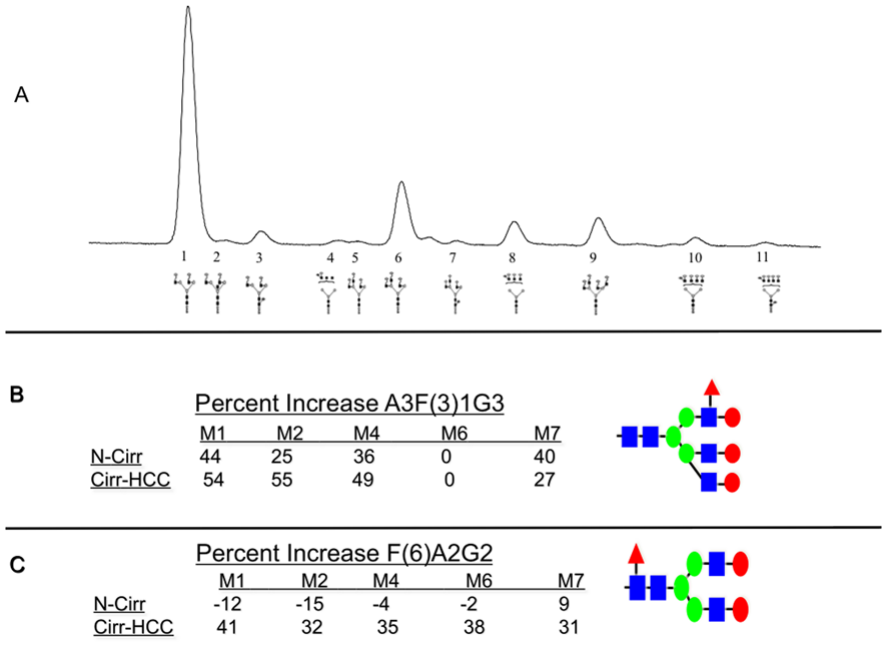
Specific changes in glycosylation on A1AT can be observed with the progression from liver cirrhosis to liver cancer. (**A**) A representative N-linked glycan profile from a normal control patient. The 11 major glycan structures identified are indicated and given a number. This number is used in Table 2 with structure names provided. The relative change in the level of the trianntennary N-linked glycan with a single outer arm fucose residue (A3F[3]G3) (**B**) and the core fucosylated bianntennary glycan (F[6]A2G2) (**C**) in normal to cirrhotic and cirrhotic to HCC is shown as percent change. As this figure shows, increases in outer arm fucosylation are associated with both cirrhosis and HCC, whereas increased core fucosylation is only observed with HCC.

To test if the increased level of tri-sialyated α-1,3 linked fucosylated outer arm fucosylated glycan (A3F(3)1G3S3) was the result of an increase in sialyation or an increase in the total amount of parent glycan, sialic acid was removed enzymatically and the sample re-analyzed. Figure 2 shows the simplified desialylated glycoprofile of the five major isoforms for each of patient group following treatment with neuraminidase (*Arthrobacter ureafaciens*). Three peaks of interest, indicated with a star in Figure 2, are reproducibly altered in the M1, M2, and M4 isoforms as the diseased liver progresses from cirrhosis to cancer. Sequential exoglycosidase digestion (data not shown) showed these peaks to be a core fucosylated biantennary glycan (F(6)A2G2), a triantennary glycan (A3G3), and a triantennary glycan with a single α 1,3 linked outer arm fucose residue (A3F(3)1G3). Figure 3A shows a representative desialylated profile with the corresponding glycan structure identified for each peak. Table 2 is a quantitation of each glycan structure present on the five major isoforms for each patient group. Specific changes in glycosylation are observed in the A1AT isoforms with the progression to cirrhosis and HCC. On M6, the isoform with very little tri and tetra-antennary structures, the biantennary core fucosylated glycan (F(6)A2G2), represents 4.48% in normal patients, 4.37% in patients with cirrhosis, and 7.04% in patients with cancer, a trend seen across each isoforms (Table 2, glycan 3). The percent change between healthy and cirrhosis and between cirrhosis and cancer is given in Figure 3C and shows that this structure changes with cancer only. In M4, the triantennary glycan with an outer arm fucose residue (A3F(3)1G3) increases from 4.34% in normal participants to 6.78% in patients with cirrhosis, and 13.18% in patients with cirrhosis plus cancer. Again, this trend is seen in each of the isoforms, with the exception of the M6 due to the total lack of triantennary structures (Table 2, glycan 8). The relative increase in each of these fucosylated glycan structures as a function of disease is shown in Figure 3B and shows that this structures changes in both liver cirrhosis and HCC. The increase in outer arm fucosylation was also associated with a decrease in the parent N-linked glycan. That is, the increase in the outer arm fucosylated triantennary glycan, A3F(3)1G3, was associated with a decrease in triantennary glycan A3G3. (Table 2, glycan 6).

**Table 2:** The N-linked Glycans Found on A1AT Isoforms from Controls, Patients with Cirrhosis, or Patients with HCC.

		M1^1^			M2^1^			M4^1^			M6^1^			M7^1^		
Glycan#^2^	Glycan Structure^4^	Healthy ^5^	Cirrhosis ^6^	HCC ^7^	Healthy ^5^	Cirrhosis ^6^	HCC ^7^	Healthy ^5^	Cirrhosis ^6^	HCC ^7^	Healthy ^5^	Cirrhosis ^6^	HCC ^7^	Healthy ^5^	Cirrhosis ^6^	HCC ^7^
1	A2G2	65.30	57.37	53.16	63.49	60.12	57.90	66.39	66.36	62.55	89.73	88.29	80.87	73.64	71.70	75.60
2	A2BG2	1.85	0.69	0.95	2.14	1.21	1.44	1.63	1.52	1.83	1.16	1.28	2.17	1.17	1.25	0.80
3	F(6)A2G2	4.05	2.89	6.09	2.19	3.73	5.54	3.62	3.47	5.30	4.48	4.37	7.04	4.62	5.11	7.36
4	A3F(3)1G(4)1	0.72	0.88	2.55	0.54	0.87	2.29	0.69	0.82	2.73	1.18	1.77	3.88	1.38	1.12	0.78
5	A3G(4,3,4)3	0.78	0.60	0.00	0.00	0.00	0.00	0.00	0.00	0.00	2.77	2.12	2.40	1.57	2.15	3.44
6	A3G3	18.70	15.44	9.62	19.26	12.72	7.65	19.83	16.29	8.65	0.28	0.65	0.69	13.04	10.13	4.74
7	F(6)A3G3	1.15	0.93	0.81	1.02	0.72	0.84	1.08	0.69	0.91	0.00	0.00	0.00	0.71	0.90	0.36
8	A3F(3)1G3	3.39	6.08	13.18	3.73	4.97	11.04	4.34	6.78	13.18	0.00	0.00	0.00	2.62	4.39	5.98
9	A4G4	2.38	7.27	2.52	4.34	7.85	2.41	0.54	0.88	0.55	0.00	0.00	0.00	0.00	0.00	0.00
10	A4F(3)1G(4)4	0.00	2.34	2.93	0.66	2.54	2.53	0.00	0.00	0.45	0.00	0.00	0.00	0.00	0.00	0.00
11	F(6)A4F(3)1G(4)4	0.00	1.22	2.78	0.00	1.46	2.27	0.00	0.00	0.00	0.00	0.00	0.00	0.00	0.00	0.00

1 A1AT isoforms as shown in Figure 1.

2 Peak number as shown in Figure 3.

3 Glycan structure as determined by exoglycosidase digestion. See text and figure 3 for more detail.

4 The percent that each glycan structure represents in the total glycan profile. The total does not equal 100% because certain minor peaks were not identified and did not alter in the patient groups.

### Analysis of Fucosylated A1AT by Lectin-FLISA in a Cohort of 458 Patients

To further examine if the changes in both core and outer arm fucosylation could be seen in individual patients and potentially used as a diagnostic marker of cancer, we analyzed a patient cohort consisting of 458 patients for the level of fucosylated A1AT using a lectin-FLISA based assay. In this assay, A1AT was captured using a monoclonal antibody and the level of fucosylation was determined using the fucose-binding AAL. AAL recognizes both outer arm and core fucosylated glycan. Twenty patients with no evidence of liver disease were used as controls; 33 patients infected with HBV had an unknown level of liver fibrosis; 215 patients infected with HCV had an unknown level of liver fibrosis; 65 patients had liver cirrhosis; 62 patients had other liver diseases; and 63 patients had liver cancer (Table 1). Figure 3A shows the relative level of fucose lectin-reactive A1AT in the six patient groups. Values are given as -fold increase in relation to the level in commercially purchased “normal” sera. The mean and 95% confidence interval of the mean are shown for each group. We found a clear statistical difference between the HCC group and all other groups (P<0.0001) and between the cirrhotic group and the control group (P = 0.0173) but not between any other groups (Figure 3A). The mean level of lectin-reactive A1AT was 1.4-fold (±0.80) above sigma in the control group, 1.7-fold (±0.1.8) in the group infected with HBV, 1.9-fold (±1.8) in the group infected with HCV, 2.6-fold (±2.3) in the group with cirrhosis, 2.6-fold (±2.0) in the group with other liver diseases, and 7.70-fold (±4.45) in the group with HCC. Surprisingly, there was no difference in the mean level of AAL-reactive A1AT in HCC patients in regards to the stage of HCC (data not shown). That is patients with stage 1 HCC, as defined as a single lesion of less than 2cm [11], had a 9.1 fold (±4.70) increase in the level of AAL reactive A1AT while patients with stage 4 HCC (those with multiple lesions >6cm in diameter) had a mean of 6.7 fold (±5.54), which was not different than that observed in stage 1 patients (*p* = 0.114).

In this cohort, fucosylated A1AT could distinguish between HCC and non-HCC cases with an area under the receiver operator curve (AUROC) of 0.871. When comparing only HCC versus cirrhosis, the discriminatory ability was 0.867. Using a cut-off of 5 relative units AAL reactive A1AT could differentiate HCC from cirrhosis with a sensitivity of 70% and a specificity of 86%. In contrast, AFP, when analyzed in this cohort, had an AUROC of 0.764 and could differentiate HCC from cirrhosis with a sensitivity of 59% and a specificity of 93% using a cut-off of 20 ng/mL.

### False Positives Have Increased Levels of Outer Arm Fucosylation Whereas Core Fucosylation Is Specific for HCC

**Figure 4:**
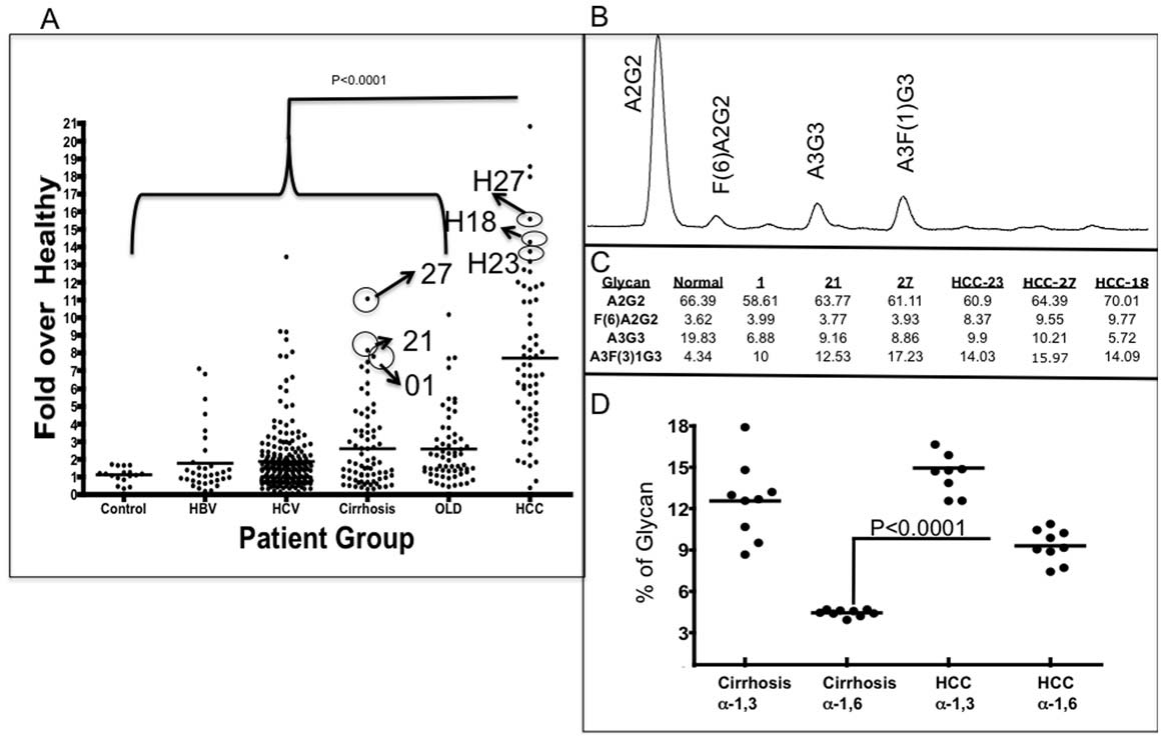
Increase in lectin-reactive A1AT with the development of HCC and identification of core fucose as a specific marker of liver cancer. (**A**) The level of lectin-reactive A1AT in patients with HCC, HBV infection, HCV infection, or other liver diseases (OLD) and in controls. The solid line represents the mean value. The x-axis represents the patient group. The y-axis shows the -fold increase in lectin-reactive A1AT compared with that in commercially purchased serum. (**B**) Glycan analysis of A1AT isoform M4 from patient 21. (**C**) Quantification of glycan analysis from three cirrhotic patients (01,21 and 27) and three patients with stage 1 or 2 HCC (HCC-23, HCC 27 and HCC 18). The levels of 4 major glycan structures are shown. As this figure shows, in cirrhotic false positives, there is an increase in outer arm but not core fucosylation. Consistent with data shown in Figures 2 and 3, increases in core fucosylation on AAT were observed only from patients with HCC. (**D**) Scatter plot of the level of α-1,3 or α 1,6 linked fucose from 9 cirrhotic false positives and 9 patients with either stage 1 or 2 HCC.

Some patients do not have cancer but have elevated levels of lectin-reactive A1AT (Figure 4A). Because we observed changes in outer arm fucosylation on A1AT from patients with cirrhosis, it was of interest to determine if those with false positive results had core or outer arm fucosylation. Figure 4A shows the results of glycan analysis from purified A1AT from three cirrhotic patients (out of nine) and three patients with stage 1 or 2 HCC (out of nine) and analyzed as before. The results from these patients are shown in Figure 4A, and a representative glycan profile of one of these patients is shown in Figure 4B, along with the level of 4 major glycan structures indicated. As Figure 4B shows, consistent with the results presented in Table 2 and in Figures 3A–3C, the three cirrhotic patients have levels of core fucosylation (F(6)A2G2) similar to those observed in healthy controls. However, these patients have significant increases in outer arm fucosylation (A3F(3)1G3), similar to the highest levels seen in patients with HCC. That is, the level of outer arm fucosylation in these patients ranged from 10% to 17%, which is similar to the level observed in patients with HCC (13%). In contrast, three patients with HCC who had very strong positive results from the lectin FLISA test had increased core and outer arm fucosylation. For example, the core fucosylated bianntennary glycan represented 9.55% on A1AT purified from patient H27. Similarly, this glycan represented more than 9% on A1AT purified from patients H18 and H23. Increases in outer arm fucosylation ranging from 14.03% to 15.97% of the total glycan on A1AT were also observed in these patients. Figure 4D shows the results of all 18 patients with a focus on the level of outer arm (α-1,3) and core (α-1,6) fucosylation. When examining all 9 cirrhotic false positives the mean level of core fucosylation was 3.77%±0.25 and the mean level of outer arm fucosyalation was 11.88%±2.79. In the case of the cancers the mean level of core fucosylation was 8.62%±1.2 and the mean level of outer arm fucosylation was 14.26%±1.9. There was statistical difference between the level of core α-1,6 fucosylation between these nine cirrhotic and nine HCC patients (p<0.0001) but not with α-1,3 linked fucosylation (p = 0.07). Importantly, the maximum level of core fucosylation observed in the false positive cirrhotics was 4.01%, similar to what was observed in healthy controls (3.62%).

## Discussion

Recent reports have indicated that increased core and outer-arm fucosylation could be observed following glycomic analysis of either total human serum or serum depleted of immunoglobulin [18], [19], [25]. We examined the glycosylation of a single protein, A1AT, as a function of liver cirrhosis and liver cancer. To this end, we found some changes that were consistent with our previous findings such as an increase in core fucosylation as well as changes in outer arm fucosylation.

The observation that changes in both core and outer arm fucosylation occur may provide clues to the molecular basis of this change. That is, although the exact mechanisms for increased core fucosylation in HCC are unknown, they are thought to involve increases in both the levels of the enzyme and the substrates involved in core fucosylation [17].

It is also possible that these markers reflect some alteration in the Golgi apparatus. Recent reports have suggested that, in regards to the liver, the fucosylation of proteins is involved in protein sorting to the bile [26]. Thus, it is conceivable that the appearance of fucosylated proteins in the serum may reflect a common defect in protein sorting. It is interesting to note that the glycosylation of A1AT in the serum of patients with liver cancer (Figures 2, 3 and 4) is similar to that observed in the bile of healthy individuals [26].

It is also interesting to note that lectin reactivity was greater than the total change observed in fucosylation. For example, patient 27 had either core or outer arm fucose on 22.82% of his or her N-linked glycans. In contrast, A1AT from a healthy individual had fucose (core or outer arm) on 9% of the N-linked glycans. Hence, using the lectin FLISA, we should have observed closer to a 2.5-fold increase and not the 11-fold increase that was obtained. This difference may be an artifact of the lectin-FLISA, the result of other proteins attached to A1AT, increased or modified O-glycosylation, or increased accessibility of the lectin to the fucose residues. All of these possible scenarios are under investigation.

It is also important to note that increased outer arm fucosylation was observed both in patients with cirrhosis and in those with HCC. This finding implies that outer arm fucosylation was not specific to cancer but rather was probably associated with inflammation, first from the liver cirrhosis and then from the presence of the HCC lesion. Indeed, the level of outer arm fucosylation may be a more specific marker of inflammation and may be predictive of cancer development [27], [28]. In contrast, increases in core fucosylation are observed only in patients with HCC, suggesting that this change is cancer specific.

As Figure 4A shows, many patients in the non-HCC groups have elevated levels of lectin-reactive AAL. Analysis of three patients with elevated AAL levels from the cirrhosis group indicated that they had changes primarily in outer arm fucosylation, not in core fucosylation. To that end, core fucosylation may be a more specific target for the detection of cancer [29]. Efforts to use lectins such as lens culinaris and *Pisum sativum* agglutinin, which will not bind outer arm fucosylated glycan, in the lectin FLISA proved problematic because these lectins have much weaker binding affinities and do not actually bind fucose directly. Efforts to make recombinant AAL with specificity only to core-linked α-1,6 fucose are currently underway.

In summary, we have analyzed the glycosylation of A1AT as a function of HCC and used a lectin FLISA to measure this change in a cohort of more than 400 patients. These data need to be confirmed in larger cohorts of patients to determine if these markers are truly reliable serum markers of early HCC, to compare their accuracy with AFP in patients of diverse gender, ethnicity, etiologies of liver disease, and to determine their role in HCC surveillance. Future studies should also test the benefit of combinatorial analysis with other potential markers of HCC, such as des-gamma-carboxy prothrombin as well as to examine how the level of core and outer arm fucosylation varies as a function of anti-cancer treatment.

## Supporting Information

Figure S1 The sialylated N-linked glycan profile for each of the five A1AT isoforms from normal, cirrhotic, or HCC patients. (A) A representative sialyated profile of the M4 A1AT isoform from healthy individuals. (B) The relative percent of sialyated bi-antennary or tri-antennary glycan in each A1AT isoform.
(3.00 MB TIF)
