# Supplementary figures and images for: Linkage Specific Fucosylation of Alpha-1-Antitrypsin in Liver Cirrhosis and Cancer Patients: Implications for a Biomarker of Hepatocellular Carcinoma
Source: PLoS One. 2010 Aug 25;5(8):e12419. doi: 10.1371/journal.pone.0012419 (PMC2928295; doi:10.1371/journal.pone.0012419)

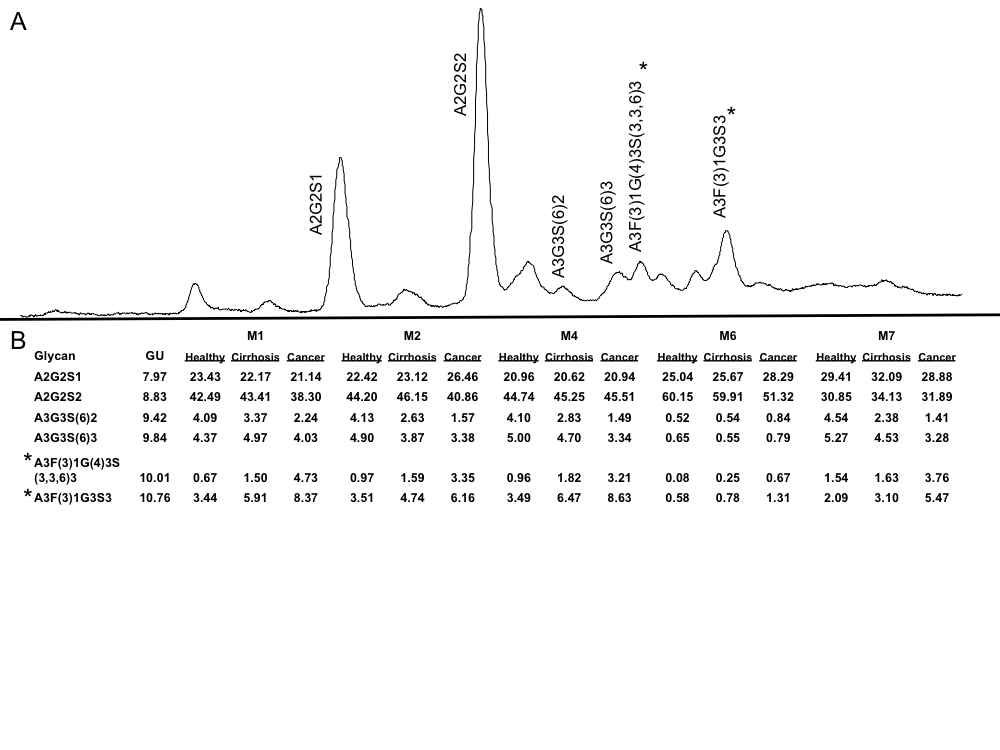

Supplement: Figure S1 — The sialylated N-linked glycan profile for each of the five A1AT isoforms from normal, cirrhotic, or HCC patients. (A) A representative sialyated profile of the M4 A1AT isoform from healthy individuals. (B) The relative percent of sialyated bi-antennary or tri-antennary glycan in each A1AT isoform. (3.00 MB TIF) [file pone.0012419.s001.tif]
